# Novel simple sequence repeats (SSRs) detected by ND-FISH in heterochromatin of *Drosophila melanogaster*

**DOI:** 10.1186/1471-2164-12-205

**Published:** 2011-04-26

**Authors:** Ángeles Cuadrado, Nicolás Jouve

**Affiliations:** 1 Department of Cell Biology and Genetics, University of Alcalá de Henares, 28871 Alcalá de Henares, Madrid, Spain

## Abstract

**Background:**

In recent years, substantial progress has been made in understanding the organization of sequences in heterochromatin regions containing single-copy genes and transposable elements. However, the sequence and organization of tandem repeat DNA sequences, which are by far the majority fraction of *D. melanogaster *heterochromatin, are little understood.

**Results:**

This paper reports that the heterochromatin, as well as containing long tandem arrays of pentanucleotide satellites (AAGAG, AAGAC, AATAT, AATAC and AACAC), is also enriched in other simple sequence repeats (SSRs) such as A, AC, AG, AAG, ACT, GATA and GACA. Non-denaturing FISH (ND-FISH) showed these SSRs to localize to the chromocentre of polytene chromosomes, and was used to map them on mitotic chromosomes. Different distributions were detected ranging from single heterochromatic clusters to complex combinations on different chromosomes. ND-FISH performed on extended DNA fibres, along with Southern blotting, showed the complex organization of these heterochromatin sequences in long tracts, and revealed subclusters of SSRs (several kilobase in length) flanked by other DNA sequences. The chromosomal characterization of C, AAC, AGG, AAT, CCG, ACG, AGC, ATC and ACC provided further detailed information on the SSR content of *D. melanogaster *at the whole genome level.

**Conclusion:**

These data clearly show the variation in the abundance of different SSR motifs and reveal their non-random distribution within and between chromosomes. The greater representation of certain SSRs in *D. melanogaster *heterochromatin suggests that its complexity may be greater than previously thought.

## Background

One of the most enigmatic aspects of genome organization in multicellular eukaryotes is the regionalization of chromosomes into euchromatin and heterochromatin domains. Heterochromatin, originally named "junk DNA" because no coding function could be found for it, is now considered essential for the epigenetic maintenance of centromeric function as well as for other cellular, developmental and evolutionary processes [1-3]. In most eukaryotes, the main components of heterochromatin are families of highly tandem repeated DNA or satellite DNA organized as multiple copies of a monomer sequence arranged in a head to tail pattern over megabase-long arrays. Running from a few base pairs to more than 1 kb in length, repeated units show a wide range of sizes and complexity. Despite sequence divergence between monomers of the same family often being very low over long arrays, satellite DNA in heterochromatin can change rapidly in nucleotide sequence or copy number during evolution. As a consequence, large numbers of unrelated satellite DNA families commonly compound the profile of satellite DNA in genomes [4,5].

*Drosophila melanogaster *provides a model for studies of heterochromatin. About 59 Mb of the 176 Mb female genome, including the proximal half of the X chromosome, the pericentromeric region of autosomes 2 and 3, and most of the dot-like chromosome 4 is heterochromatic. The entire male Y chromosome (41 Mb) is also heterochromatic [6]. However, polytene chromosomes, which have proved useful in mapping euchromatin regions, provide minimal resolution in heterochromatin analyses. Due to its late replication, the heterochromatin remains immersed in the diffuse and unbanded chromocentre region. Thus, mitotic rather than polytene chromosomes are preferable for chromosome mapping of heterochromatic sequences [7].

Over the last three decades, different families of highly repeated tandem sequences have been molecularly characterized and physically mapped to the chromosomes of *D. melanogaster *using different cloning strategies. Most of the highly repeated families of the fruit fly genome are composed of short repeat units 5-12 bp long, arrayed in tandem and extended over several megabases of DNA. These highly repeated sequences are localized in specific segments of the heterochromatin, which may contain different sets of satellite DNA [8-11].

The goal of large-scale genome projects is to convert the initial draft of euchromatic sequences into a complete telomere-to-telomere sequence for each chromosome. The Drosophila Heterochromatin Genome Project has made substantial progress in identifying contiguous sequences of the heterochromatin regions containing single-copy genes and dense clusters of transposable elements. However, the sequence and organization of the highly tandem repetitive DNA fraction - the vast majority of *D. melanogaster *heterochromatin - is little known [12]. The apparent absence of identified highly repeated sequences in certain heterochromatic regions and BACs covering the extensive heterochromatin gaps, suggests that unknown classes of highly repetitive DNAs must be present in this fraction of the *D. melanogaster *chromosomes. To determine what they are will require new technologies [13].

Short motifs of 1-6 bp repeated in tandem are classified as SSRs or microsatellites [14,15]. Although the genomes of higher organisms contain some long microsatellites (up to 500 nucleotides), in general these polymorphic loci are no longer than 100 nucleotides. However, in many species, SSRs may also be organized into long stretches of nearly 100 to several thousand tandem units mainly clustered in the heterochromatin, also referred to as satellite SSRs [16,17]. For example, in many vertebrate species, the heterochromatic sex chromosomes are rich in clusters of GATA and GACA repeats [18]. Long arrays of AAGAG and AATAT are found in the heterochromatin of fruit fly chromosomes [10]. Moreover, GGAAT and CATTT repeats have been found in the satellite regions of human chromosomes [19]. There is some evidence that the origin and evolution of satellite families, including those with relatively complex and longer repeated units, can be explained by the autoreplicative properties of different SSRs [20].

In the present study, a recently reported, simple and highly efficient non-denaturing FISH method (ND-FISH) was used to localize the classic satellites (AAGAG, AAGAC, AATAT and AATAC) and AACAC repeats [21]. Heterochromatic regions enriched in SSRs were also sought, and the physical distribution of 16 different SSR types (mono-, di-, tri- and tetranucleotide repeats) studied in both polytene and mitotic chromosomes. An ND-FISH analysis employing DNA fibres was also performed and the SSRs characterized by Southern hybridization.

## Methods

### Chromosome preparation

Mitotic and polytene chromosomes of *D. melanogaster *were obtained using third instar larvae of a wild type strain kept in our laboratory for about 20 years. The brains and salivary glands of individual larvae were dissected out in 45% acetic acid before squashing on a clean microscope slide with a drop of 45% acetic acid. After removing the cover slips by freezing, the slides were air dried.

DNA fibres were obtained from disrupted nuclei following a derivative of the alkaline/ethanol method described by Fidlerová et al. [22]. The brains of third instar larvae were dissected in a drop of water. Neuroblast tissues was disaggregated with a pestle in a drop of 3:1 ethanol:acetic acid on one end of a clean microscope slide. The resulting cell suspension was allowed to air-dry. The slides were then rinsed in 100 ml of phosphate-buffered saline solution (PBS) in a Coplin jar for 1 min. After removing the PBS solution (but not allowing the slide to dry out), the cell-containing area was incubated with 100 μl of lysing buffer (0.5 M NaOH/30% ETOH) for 1 min to disrupt the nuclei before stretching the fibres by tilting the slide at 45° and letting the buffer run away. 100 μl of 100% ethanol were then added to the cell-containing area before post-fixation employing an ethanol series (70%, 100%) (performed in a Coplin jar), allowing 5 min in each solution. Finally, the slides were air-dried.

### Probes and labelling

Table 1 shows the twenty one oligonucleotide probes used. These were obtained from different companies, and had biotin and/or digoxigenin incorporated at their ends.

**Table 1:** Oligodeoxyribonucleotides used as probes to analyze SSRs in the present study and references of works where the distribution of SSRs has been reported previously by in situ hybridization in *D. melanogaster *chromosomes.

SSRs	Probes used in study	References	
		
		Mitotic chromosomes	Polytene chromosomes
A	A_20_		(21, 40)

C	C_20_		(40)

AC; AG	(AC)_10_; (AG)_10_	(27)	(27, 40)

AAC; AAG; AAT, AAC; AGC; ACT; AGC; AGG; ATC; CCG	(AAC)_5_; (AAG)_5_; (AAT)_5; _(ACC)_5_; (ACG)_5; _(ACT)_5; _(AGC)_5_; (AGG)_5_; (ATC)_5; _(CCG)_5_		

GACA	(GACA)_5_		(21)

GATA	(GATA)_5_	(31)	(21)

AAGAC; AAGAG; AATAC; AATAT	(AAGAC)_3_; (AAGAG)_3_; (AATAC)_3_; (AATAT)_3_	(10)	

AACAC	(AACAC)_3;_	(11)	(11)

### Non-denaturing fluorescence in situ hybridization

ND-FISH was performed as previously described [21]. Briefly, preparations were incubated at 24°C in a humidity chamber for 2 h with 30 μl of hybridization buffer containing 2 pm of the probe in 2×SSC. For post-washing, slides were immersed in 4×SSC/0.2% Tween^20 ^and agitated for 10 min at room temperature (RT). The detection of digoxigenin and biotin was respectively undertaken by incubating the slides in fluoresceinated anti-digoxigenin (Roche Applied Science) or streptavidin-Cy3 (Sigma) in 5% (w/v) BSA for 1 h at 37°C. Before staining the DNA with DAPI, the slides were rinsed for 10 min in 4×SSC/0.2% Tween^20 ^at RT. The slides were then mounted in antifade solution (Vector Laboratories).

### Fluorescence microscopy and imaging

Slides were examined using a Zeiss Axiophot epifluorescence microscope. For each motifs we analysed at least 10 cells. The images from the biotin/Cy3, digoxigenin/Fitc and DAPI staining procedures were recorded separately using a cooled CCD camera (Nikon DS). The exposure times were variable depending of the intensity of the *in situ *signals obtained with each probe. The localization of the signals relative to the DAPI banding pattern was resolved by merging digital images using Adobe Photoshop. The length of single DNA fibres was measured taking into account that a beaded, interrupted signal often denotes a maximally decondensed DNA fibre. An average of 3 Kb μm^-1 ^is reported for mammalian and *Drosophila *DNA [23,24].

### Southern blot analyses

Genomic DNA was isolated from adult flies using the DNeasy Blood & Tissue kit (Qiagen). DNA from both males and females (7.5 μg) was digested with *Alu*I, *Rsa*I and *Hinf*I restriction endonucleases, divided into three aliquots and separated on 1% agarose gel. After electrophoresis the gels were blotted onto nylon membranes (Biodyne, Pall Corporation) under alkaline conditions following standard techniques. Each membrane was cut into three and hybridized successively with different digoxigenin-labelled oligonucleotides as probes. To ensure high stringency, the temperature of hybridization and the post-hybridization washes were estimated for each oligonucleotide probe according to the Tm and adhering to the GC content rule: 30.7°C for AATAC_3_, 36.2°C for ACT_5_, AAG_5 _and AAC_5_, 38.9°C for AAGAG_3_, AACAC_3 _and AAGAC_3_, 44°C for GATA_5_, and 54.3°C for AG_10_, AC_10 _and GACA_5. _Hybridization was performed overnight using 2 pm of oligonucleotides in 5×SSC, 0.02% SDS, 0.01% LSS and 0.3% blocking reagent (Roche). Filters were washed with 0.15×SSC and 0.1% SDS at the corresponding Tm for 30 min before detection using anti-digoxigenin-AP, Fab fragments (Roche), and CDP-Star (Roche) according to the manufacturer's recommendations. The techniques followed were basically as described in Loarce et al. [25].

## Results

### Chromosomal localization of pentanucleotide DNA satellites by ND-FISH

**Figure 1:**
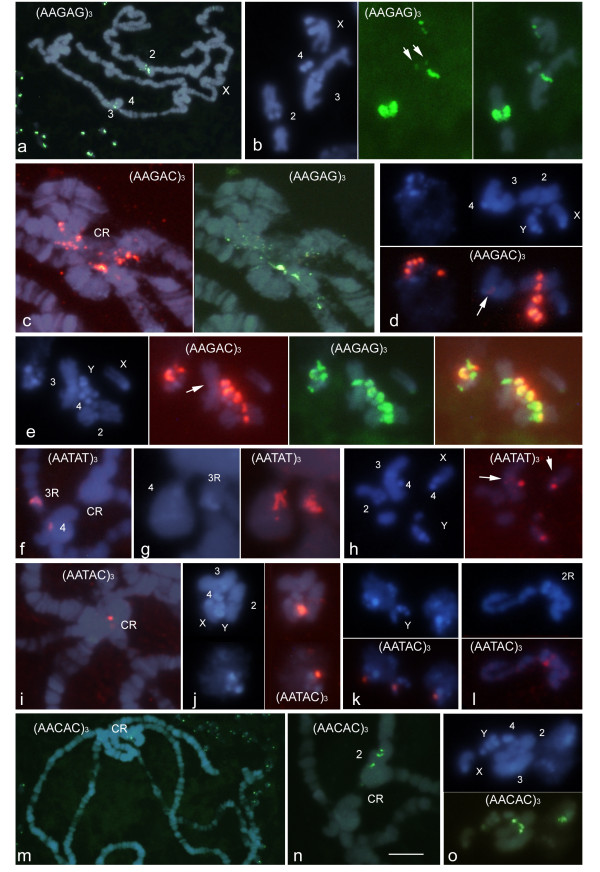
**Photomicrographs showing the motif-dependent chromosomal distribution of the pentanucleotide probes in the salivary gland (a, c, f, g, i, m and n) and neuroblast cells (a, b, d, e, h, j, k, l, m and o) of male and female *D. melanogaster *after ND-FISH and DAPI staining**. Each panel shows individual and/or merged images to facilitate the visualization of the signals (green or red for digoxigenin- and biotin-labelled probes respectively) with respect to the DAPI (blue) banding pattern. In some samples interphase and mitotic neuroblast nuclei are shown in the same panels; both polytene and diploid nuclei are shown in **a **and **m**. Mitotic chromosomes are identified as well as polytene regions of interest. An example of two-colour ND-FISH is shown in **e**. Note that only the diffuse chromocentre (CR) observed in polytene nuclei is enriched in pentanucleotide SSRs, which localize to specific heterochromatic regions in mitotic chromosomes as shown in Figure 4. The arrows point to low intensity signals. Scale bar: 5 μm, except in **a **and **m**, in which it represents 25 μm.

To verify whether ND-FISH was a suitable technique for identifying clusters of SSRs in the heterochromatin of *D. melanogaster*, the chromosomal distributions of five pentanucleotide probes were analyzed: (AAGAG)_3_, (AAGAC)_3_, (AATAT)_3_, (AATAC)_3 _and (AACAC)_3 _(Figure 1). These probes were chosen because they share up to 80% homology and represent well characterized families of satellite DNA, which differ in abundance, complexity and chromosomal distribution. The neuroblasts and salivary gland cells obtained from individual larvae (squashed on the same slide) were analysed. This allowed the distribution and intensity of the *in situ *signals obtained simultaneously with each probe in both diploid and polytene nuclei to be compared (Figure 1a and 1m).

The AAGAG probe was found at different intensities in multiple clusters in the chromocentre of polytene chromosomes and in interphase neuroblast nuclei (Figure 1a-c and 1e). All mitotic chromosomes showed *in situ *signals (Figure 1b and 1e). The number of clusters on chromosome 2 could not be determined due to the proximity of their signals, which were arranged in a dispersed pattern along most of the heterochromatin (het) of the right arm (2Rhet). The multiple sites observed on the Y chromosome were concentrated in three regions along its length, with two on YL and one on Y^S^. AAGAG repeats were also found in significant amounts on 3Rhet and in two minor clusters, one distal on XR and the other on chromosome 4.

(AAGAC)_3 _showed a similar chromosomal distribution to (AAGAG)_3_. However, when two-colour ND-FISH was performed using both probes simultaneously, differences were observed between the polytene and mitotic chromosomes (Figure 1c and 1e). In male metaphases, as with the AAGAG probe, multiple sites were observed on the Y chromosome, concentrated in the same regions (Figure 1e). The AAGAC repeats were localized in a strong cluster on 2Rhet and in a minor site on 3Rhet (Figure 1d).

The AATAT probe was also found in the chromocentre of polytene chromosomes (Figure 1f). A detailed analysis allowed the AATAT repeats to be localized precisely at the base of the polytenized regions of chromosomes 3R and 4 (Figure 1g). In mitotic chromosomes these repeats were primarily found on chromosome 4. In addition, multiple sites of lower intensity were detected on chromosome Y (the strongest at the two tips). Two minor clusters were also seen, one distal on XR and the other on 3R, presumably near the euchromatin-heterochromatin junction (Figure 1h).

The AATAC probe was detected in the chromocentre of polytene chromosomes (Figure 1i). In mitotic chromosomes, a strong cluster on YL (Figure 1j-k) and a minor site on 2Rhet were observed (Figure 1l).

Finally, a few AACAC signals were found dispersed in the chromocentre (Figure 1m), in the unbanded regions of chromosome arm 2R (Figure 1n). In metaphase chromosomes these repeats were situated on 2Rhet and Y^S ^(Figure 1o).

### Chromosomal localization of mono- and dinucleotide SSRs by ND-FISH

**Figure 2:**
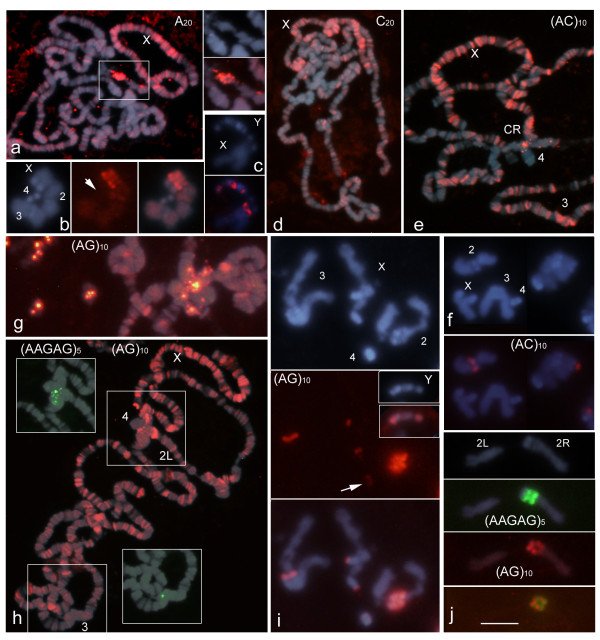
**Chromosomal distribution of the mono- and dinucleotide SSRs in the salivary gland (a, d, e, and h) and neuroblast cells (b, c, f, i and j) of *D. melanogaster *after ND-FISH and DAPI staining**. Both polytene chromosomes and diploid nuclei are shown in **g; **examples of two-colour ND-FISH with (AAGAG)_5 _are shown in **h **(insets) and **j**. Each panel shows individual and/or merged images to facilitate the appreciation of the distribution of signals (green or red from digoxigenin- and biotin-labelled probes respectively) and identification of chromosomes. The localization of the signals with respect to the heterochromatic DAPI banding pattern in mitotic chromosomes is shown in Figure 4. Note the high concentration of A, C, AC and AG SSRs on polytene X chromosomes; this contrasts with the lesser presence of these repeats on chromosome 4. An enlarged view of the A repeat signals at the base of chromosome X (square) and chromosome 4 is shown at the right of panel **a**. The arrows point chromosomes 4; CR, chromocentre. Scale bar = 5 μm and 25 μm in (pro)metaphase and polytene nuclei respectively.

Figure 2 shows the distribution of the homonucleotide probes A_20 _and C_20_, which detect arrays of A/T and C/G mononucleotides respectively as well as two alternating nucleotide repeats: (AG)_10 _and (AC)_10_. The analysis of the other two possible dinucleotide repeats, AT and CG, could not be performed accurately using hybridization techniques owing to their self-complementing.

The A_20 _probe showed a great number of signals in polytene chromosomes. A high density of A repeats was seen on the X chromosome, especially concentrated near the chromocentre (Figure 2a), as previously reported (Table 1, [21]). In mitotic chromosomes, A_20 _was found interstitially along chromosome arm XL and in three clusters on chromosome Y, with two on the long arm and one in its satellite (Figure 2c). A disperse distribution of A signals was observed throughout the euchromatin in mitotic chromosomes when using long image capturing exposure times. The highest concentration was seen on the distal half of chromosome X, contrasting with the absence of signals on chromosome 4. In addition, higher concentrations of dispersed signals were observed in the proximal chromosome arm 2R (Figure 2b).

Polytene chromosomes hybridized weakly with the C probe. However, with the help of the CCD camera, these repeats were observed as many bands that decreased in number towards the chromocentre, and which were particularly concentrated on the X chromosome (Figure 2d).

With the exception of chromosome 4, a large number of intense signals for (AC)_10 _were seen along the length of the polytene chromosomes arms. As described above for the mononucleotide repeats, the density of signal along the X chromosome was higher. In addition, a few signals of different intensity were found dispersed in the chromocentre, some in the unbanded regions of chromosome 2 (Figure 2e). In mitotic chromosomes, weak signals were seen dispersed over most of the euchromatin. A cluster of stronger signals was seen near the centromere of chromosome arm 2R (Figure 2f).

The distribution and intensity of the (AG)_10 _signals fit the general pattern described for AC repeats in polytene chromosomes (Figure 2g-h). In addition, the AG probe was found in multiple clusters of different intensity in the chromocentre. The unbanded region of chromosome 2 was particularly rich (Figure 2g-h). AG repeats were detected in multiple clusters in all mitotic chromosomes. Several sites were observed on chromosome 2, especially concentrated on 2Rhet (Figure 2j), and in the two tips of chromosome Y. A cluster was also found on 3Rhet along with two minor sites on chromosome 4 and another on the tip of chromosome XR (Figure 2i).

### Chromosomal localization of tri- and tetranucleotide SSRs by ND-FISH

**Figure 3:**
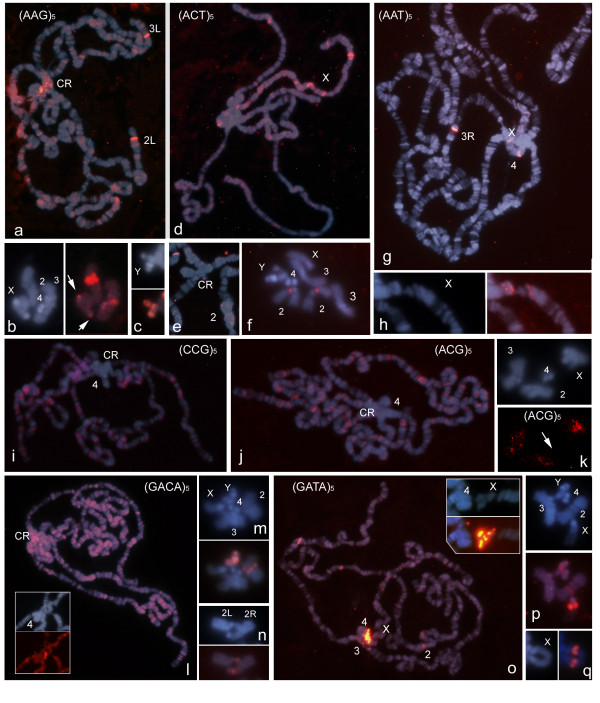
**The non-random chromosomal distribution of several tri- and tetranucleotide SSRs in polytene (panels a, d, e, g, h, i, j, l and o; scale bar 25 μm) and mitotic (b, c, f, k, m, n, p and q; scale bar 5 μm) nuclei of *D. melanogaster*, as shown by ND-FISH with the indicated biotinylated probes**. Each panel shows individual and/or merged images to facilitate the visualization of the red signals with respect to the DAPI (blue) staining and to allow chromosome identification. The view of the localization of the AAT repeats in the base of chromosome X is enlarged in **h**. A detailed view of the chromocentre (CR) after ND-FISH with (ACT)_5_, (GACA)_4 _and (GATA)_4 _is shown in **e **and in the inserts in panels **l **and **o **respectively. The arrows point to chromosomes X and 4 in **b **and **k **respectively.

Probes with all the possible trinucleotidic combinations were analysed by ND-FISH. Well-defined signals were observed only for (AAG)_5_, (ACT)_5 _and (AAT)_5_, which appeared restricted to a few unambiguous sites on specific arms of polytene chromosomes (Figure 3a, d and 3g respectively). The AAG and ACT repeats gave signals clustered in the chromocentre (Figure 3a and 3d respectively). In mitotic chromosomes, (AAG)_5 _hybridized at three sites on chromosome arm 2Rhet, two on YL and one on Y^S^. In addition, a less intense site was observed on 3Rhet and two small sites were seen on chromosomes 4 and X (Figure 3b-c). ACT repeats were exclusively found on 2Rhet (Figure 3f). The most intense AAT signals were seen in the interstitial regions of chromosome 3R and on chromosome 4, and near the chromocentre of chromosome X in polytene chromosomes (Figure 3g). A detailed analysis using high quality chromosome spreads showed clusters near the base of chromosome X (Figure 3h).

Increasing the CCD camera exposure time revealed specific patterns of low intensity signals for AAC, ACC, ATC, ACG, AGC, AGG and CCG distributed as a large number of signals along the length of the polytene arms (Figure 3i and j). Regardless of differences in the abundance and localization of each motif on the polytene chromosomes, a uniform pattern of distribution was observed over all chromosome arms, but particularly on chromosome X. The use of very long exposure times revealed a characteristic dispersion pattern of signals in mitotic chromosomes (Figure 3k).

ND-FISH was used in previous work to determine the distribution of (GACA)_5 _and (GATA)_5 _in polytene chromosomes of *D. melanogaster *([21] and Figure 3l and 3o respectively). That analysis was here extended by analysing mitotic chromosomes (Figure 3m-n and 3p-q respectively). In addition to the high concentration of GACA repeats found in the chromocentre of polytene chromosomes, a rich pattern of signals of similar intensity was seen over the chromosome arms (Figure 3l). The strongest (GATA)_5 _signals were detected at the chromocentre and a few specific sites on the chromosomes arms (Figure 3o). GACA repeats were especially numerous on mitotic chromosomes 2Rhet and Y (two clusters on YL and one site on its satellite). A minor cluster was also detected on chromosome 3Rhet. The strongest GATA signals were seen on the long arm of chromosome X, with minor sites on chromosomes 2, 4 and Y (Figure 3p-q).

### Location of SSRs relative to cytogenetic heterochromatic regions

**Figure 4:**
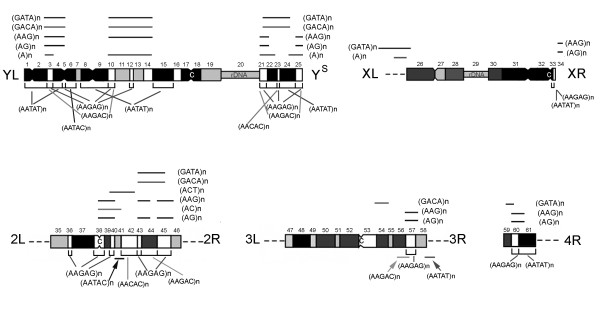
**Distribution of several SSRs in the *D. melanogaster *cytogenetic reference map of the heterochromatic regions (h1 to h61) derived from Pimpinelli et al**. [26] (the darker the block, the brighter the DAPI intensity; centromeres [C] and rDNA are also indicated). At the resolution in Figure 1, the distribution of the pentanucleotide repeats is the same as reported by Lohe et al. [10] and Mukani et al. [11]. Thus, the localizations of these pentanucleotide SSRs are indicated below the chromosomes as previously mapped. Additional localizations to those reported are shown by arrows. Lines above chromosomes represent the novel SSRs described in the present work (Figs. 2 and 3). Note that some sequence has been mapped to chromosome regions defined by several heterochromatic bands, but neither the physical size nor intensity of the fluorescence signals is represented here.

Figure 4 shows the chromosomal distribution of all the SSRs in the heterochromatin of *D. melanogaster *using a map adapted from Pimpinelli et al. [26] which recognizes 61 cytogenetic regions by DAPI banding. The localization of the AAGAG, AAGAC, AATAT and AATAC repeats agrees with that shown in the map of Lohe et al. [10], while the distribution of the AACAC repeats agrees with the map produced by Makuni et al. [11] Although several clusters of different repeat motifs were mapped to the same cytogenetic regions, the last two groups of authors were able to assign SSRs to segments of the heterochromatin using identified chromosome rearrangements and breaks in both the euchromatin and heterochromatin. With the exception of two additional clusters of AAGAC on 3Rhet and AATAC on 2Rhet, the localization of the pentanucleotide probes here analysed by ND-FISH are in good agreement with the results of the above authors, both in terms of appearance and chromosomal position (compare Figure 1 and 4). Two-colour ND-FISH clearly resolved the proximal-distal order of two probes with respect to the position of fluorescence signals within the cytogenetic map (Figure 4). For example, the previously unmapped clusters of AAGAC repeats found on 3Rhet co-localized to a proximal position with respect to a cluster of AAGAG, precisely mapped on segment h57 (Figure 1e). In the same way, the AATAC cluster detected on 2Rhet was proximal to AACAC mapped in h42, presumably in region h41. This strategy was used to assign the remaining SSRs to heterochromatin segments.

The detailed analysis of polytene chromosomes allowed the SSRs clustered at the boundaries of the pericentromeric heterochromatin to be assigned to different heterochromatin segments. For example the AATAT repeats found near the chromocentre on 3R might correspond to the signal found on 3Rhet near the frontier between the euchromatin and heterochromatin, presumably at the boundary of region h58 (Figure 1f). In the same way, the GATA repeats found near the chromocentre at the base of chromosome X might correspond to the signals clustered on the long arm of chromosome X at the heterochromatin-euchromatin border in the region h26 (Figures 3o).

### Analysis of DNA fibres by ND-FISH

**Figure 5:**
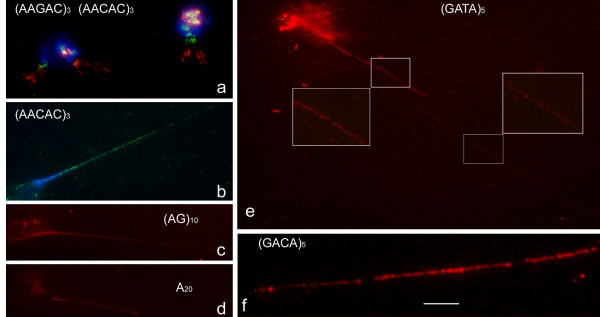
**Organization of representative SSRs in extended DNA fibres from neuroblast nuclei of *D. melanogaster *after ND-FISH with the indicated probes (green or red signals from digoxigenin- and biotin-labelled probes respectively) and DAPI (blue) staining**. An example of two colour fibre ND-FISH in haloed nuclei with (AAGAC)_3 _(red) and (AACAC)_3 _(green) is shown in **a**. Examples of fluorescent signal patterns in fibres stretched to different degrees are shown in **e **(square insets). Note the characteristic beaded nature of the signals in the highly extended fibres. A representative of the gaps observed between the continuous track of beaded signals is amplified in **f**. Scale bar = 10 μm, except in **f **in which it represents 5 μm. Every micrometer represents 3 kb of the highly stretched DNA.

To better estimate the size and molecular organization of the novel SSR clusters in the heterochromatin, the extended chromatin fibres were studied. In initial experiments, the lysed nuclei of neuroblasts were used to examine the quality of the fibres (in this case not extended) and the possibility of using ND-FISH (Figure 5a). The hybridization signals were clear for the intact nuclei still condensed and the expanded DNA around the lysed nuclei. The resolution achieved for the disrupted nuclei was sufficient to co-localize different probes as non-intermingled adjacent blocks, even when they hybridized at the same chromosome positions. Further, when extended chromatin fibres from lysed nuclei were generated, ND-FISH revealed long arrays of fluorescent signals for different probes (Figure 5b to 5f). Condensed chromatin fibres appeared as thick continuous lines; thin fibres, observed as beaded, interrupted signals, are the result of maximally decondensed DNA fibres (Figure 5e). With both the classic pentanucleotide satellites and the other SSRs found clustered in heterochromatin, fibres with comparable lengths and similar beaded patterns were observed. Assuming an average of 3 kb μm^-1 ^for the extension of maximally stretched fibres, the largest fibres observed (extended over the field of view of the 100× objective) contained several hundred kilobases. These fibres commonly presented gaps of thousands of base pairs without hybridization flanked by regions showing fluorescent signals (Figure 5f).

### Molecular organization of SSRs in *D. melanogaster*

**Figure 6:**
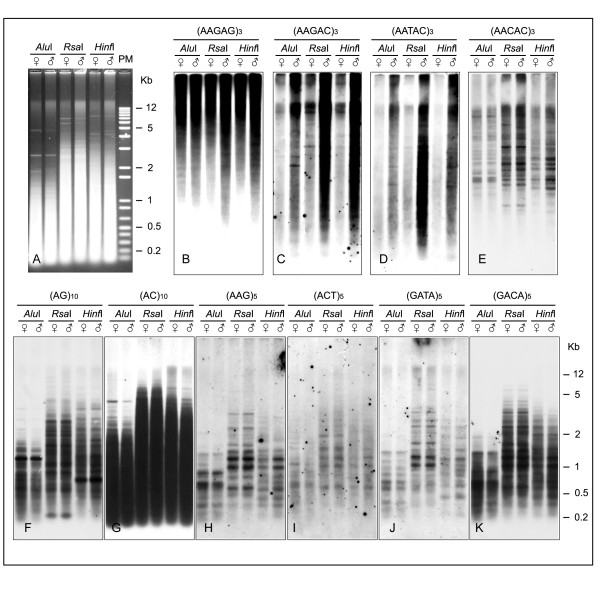
**Southern hybridization patterns of genomic DNA from female and male *D. melanogaster *digested by *Alu*I, *Hinf*I and *Rsa*I and fractionated by conventional 1% agarose gel electrophoresis (**A**)**. Three filter transfers were sequentially hybridized with the indicated digoxigenin end-labelled SSR probes (**B **to **K**). Size markers (M) are given in kb.

The molecular organization of the SSR clusters was studied by Southern hybridization. Tests were performed using DNA probes labelled with digoxigenin and genomic DNA from male and female adult flies digested separately with the restriction enzymes *Alu*I, *Hinf*I and *Rsa*I (Figure 6). The three restriction enzymes have specific recognition sites of four base pairs and digested most of the genomic DNA into fragments shorter than 1 kb. DNA resistant to the endonucleases was observed at the exclusion limit in gel electrophoresis. Different patterns of fragments were revealed for the male and female genomic DNA (Panel A). The three endonucleases used had no recognition sites within the repeat sequences analysed, allowing the identification of the full SSR tracks and short flanking sequences.

Initially our Southern results show the abundance and size of the fragments to vary significantly between different SSRs. Only the pentanucleotide probes hybridized to the restriction enzyme-resistant DNA (Figure 6B-E), indicating the absence of the restriction sites and the existence of large arrays. Three noticeable differences were detected among the probes. (AAGAG)_3 _gave the strongest hybridization pattern with the genomic DNA of both sexes, with most of the fragments longer than 20 kb (Figure 6B), while a prominent male-specific hybridization pattern was observed with the (AAGAC)_3 _and (AATAC)_3 _probes (Figure 6C-D), defining the existence of the Y chromosome. Finally (AACAC)_3 _revealed multiple and well defined bands of hybridization ranging in size from 1 to 20 kb.

The remaining SSR probes returned specific patterns of hybridization varying in number and fragment size. The (AC)_10 _probe showed the strongest hybridization, with fragments ranging from a few base pairs to 5 kb in size (Figure 6F). (AG)_10_, (AAG)_5_, (AAC)_5, _(ACT)_5 _(GATA)_5 _and (GACA)_5 _showed specific patterns with wide spectrums of well-defined fragments ranging from 0.1 to 4 kb in size (Figure 6G-K respectively).

The remarkable differences between the motifs and the composite SSRs, for example AG and AAG and the composite AAGAG or AC and AG and the composite GACA, revealed by southern pattern (Figure 6), provide evidence for the specific amplification of each SSRs at particular chromosomal locations.

## Discussion

### Reliability of the ND-FISH technique in the visualisation of SSR-enriched chromosome regions

The five pentanucleotides SSRs used to analyze the classic DNA satellites of *D. melanogaster *differ from one another by at least one nucleotide. Therefore, it is to be expected that these probes might hybridize with one another under low stringency hybridization conditions. The contrasting distribution results for these SSRs show, however, that even probes with 80% identity provide motif-specific hybridization patterns in ND-FISH. Moreover, except for two faint additional signals with (AATAC)_3 _and (AAGAC)_3_, the results obtained here were in complete agreement with previous localizations involving clones containing the satellite repeats as probes in high stringency *in situ *hybridization experiments using other flies stocks ([10,11] and Figures 1 and 4). These results suggest that heterochromatic SSRs clusters are conserved among strains. However we can no discard certain level of polymorphism (extents/distributions of SSRs) among different flies stocks. Additional clusters of AG and AC on mitotic chromosomes were detected beyond those discovered in previous work involving FISH and long duplex probes composed of simple motifs [27]. This result indicates that ND-FISH is a higher efficient technique improving the sensitivity of detection of SSRs enriched chromosome regions.

No attempt was made to construct a higher resolution physical map of SSR clusters in the heterochromatin. Even when analysing the heterochromatic regions in well spread prometaphase cells, it was impossible to reliably assign probes to specific blocks in DAPI-stained chromosomes (since there are about 100 Mb of heterochromatin divided into 61 bands) (e.g., see Figure 1b and 2i). Further, due to the fluorescence spreading, it could not be determined whether strong signals were actually produced by two or more neighbouring SSR clusters. Nevertheless, Figure 4 provides a useful working map of the highly repetitive components of the *D. melanogaster *genome, which further analyses ought to improve (e.g., using simultaneous and/or sequential ND-FISH with different probes and analysing ordered chromosome rearrangements or chromosomes with deletions of specific heterochromatic bands, etc.).

The ND-FISH technique is simpler, faster and more efficient than any previously reported in situ method for analyzing the chromosome distribution of SSRs [21]. Another advantage of ND-FISH over standard FISH is that it avoids chromosome denaturation; thus, the morphology of the chromosomes is well maintained. This is very important when successive reprobing is required on the same slide. Moreover, ND-FISH probably prevents DNA losses. This is desirable for higher resolution mapping using DNA fibres, which often become fragmented during the denaturing step of standard FISH. Thus, ND-FISH resolves one of the main limitations of the analysis of DNA fibres by conventional FISH. The results in Figure 5 show that neuroblasts are an excellent source of nuclei for lysing, and that oligonucleotides used as probes in ND-FISH permit the mapping of SSRs in extended chromatin fibres.

### *D. melanogaster *heterochromatin is enriched in SSRs

*D. melanogaster *is a model for heterochromatin studies and the euchromatin-heterochromatin boundaries have been fully analysed. Moreover, heterochromatic sequences of about 20 Mb, mainly containing single-copy sequences and transposable elements, have been almost completely mapped [13,28]. However, as in other "finished" genomes, the sequence and structure of the main components of the heterochromatin, which contains highly repetitive tandem sequences, are poorly characterized [29].

As far as we know, the only SSRs molecularly characterized with a unit length under 4 bp in the heterochromatin of *D. melanogaster *are TAA and GATA. TAA is involved in a complex sequence of 5.9 kb which maps to h26, the most distal division of the cytogenetic map [30]. The present data show a distribution of AAT very close to the proximal region of chromosome X (Figure 3h), suggesting it might be the same sequence array.

Singh et al. [31] reported the existence of GATA repeats concentrated in the proximal region of the X chromosome as a minor repeat class in the genome of *D. melanogaster*. The sequencing of chromosome X showed GATA to also be present in the pericentromeric heterochromatin ends while the most proximal sequence scaffold begin also in TAGA repeats, flanking a gap of unknown size in the heterochromatic region h26 [13]. The present ND-FISH data suggest that the stronger signals found with the (GATA)_5 _probe at the euchromatin-heterochromatin boundary of the X chromosome (Figure 3o-q) might represent this unsequenced gap.

### Structure of SSRs clusters

Satellite DNA can be extended over megabases of DNA but the maximum length is unknown due to the lack of large DNA clones containing highly repeated sequences. For example, two large blocks of AATAT and AAGAG satellites ranging up to approximately 380 kb, interrupted by five transposons, have been identified in the functional centromeric region of the minichromosome Dp1187 in *D. melanogaster*, but only contigs of about 8 kb of these repeats have been obtained [32,33].

Several kilobases of target sequence are required to produce consistent hybridization signals in metaphase chromosomes, although this does not mean that the target must exist in long interrupted stretches. On the other hand, no important differences were found between the probes here analysed for visualising the arrays of SSRs using DNA fibres (Figure 5). The characteristic appearance of a beaded rather than a continuous fluorescent track limits the estimation of the number of repeats in the continuous tandem. Thus, in addition to the gaps observed, suggests several kilobases of unrelated sequence flanked by SSRs, the existence of several DNA kilobases of unrelated sequences within the fluorescence string of signals cannot be ruled out (Figure 5f).

Clear differences were observed after Southern blot hybridization. The pentanucleotides, found exclusively in the heterochromatin by ND-FISH, hybridized at the exclusion limit of the gel, indicating the existence of large arrays of these repeats in the enzyme-resistant DNA fraction (Figure 6B-E). In addition, the specific pattern of multiple fragments observed over a wide range of molecular weight probably reflects heterogeneity within the tandem repeats and/or the existence of other sequences present within these highly repetitive blocks. This agrees with the analysis of sequences of individual pentanucleotide SSR tracts, demonstrating that they could be imperfect and might be flanked by DNA sequences, mainly related to transposable elements [8,33].

None of the SSRs with a repeat motif of less than four bases analysed by Southern blotting hybridized with the undigested DNA fraction, and no fragments with a molecular weight of over 10 kb were found (Figure 6F-K). This suggests that these SSRs must be organised in clusters of short tandem repeat stretches instead of the longer blocks that characterize the classic satellites. However, they provide the extended regions of several kilobases required to produce consistent *in situ *signals on mitotic chromosomes. The differences in genomic organisation suggested by the Southern blot analysis might explain why the novel SSRs clustered in the heterochromatin of *D. melanogaster*, have never been previously isolated or cloned using the satellite DNA fragments obtained by ultracentrifugation in caesium density gradients [8].

### Abundance and distribution of SSRs in *D. melanogaster *chromosomes

The analysis of complete euchromatin sequences revealed that, as for most of the genomes examined to date, the *D. melanogaster *genome shows great variation in terms of the abundance and distribution of SSRs [34-37]. The present results for the polytene chromosomes agree with some of the above database-held information. For example, in the euchromatic chromosome arms, the SSRs based on the dinucleotides AC and AG were the most common, followed by the SSR with the mononucleotide A. In general, trinucleotide repeats were only detected in small amounts by ND-FISH (Figure 3a-k). These results might be explained by the size of the SSR loci and the abundance of the different motifs found in *D. melanogaster *genome. The frequency of the complete trinucleotide SSR set was found to be about half that of the AC repeat loci. Moreover, about 10% of the AC repeat loci have more than 100 bp while trinucleotide SSRs loci with more than 50 bp are very rare and only the ACT and AAT repeats have more than 100 bp [38]. Therefore, a minimum target sequence size (long SSRs or high density of short loci) is required for ND-FISH signals to be detected even when using polytene chromosomes. As expected, because long pentanucleotide SSR loci are rare in *D. melanogaster *sequenced genome they were mainly localized in the chromocentre of polytene chromosomes (Figure 1). However, half of the SSRs scanned in this work, some of which were abundant in the heterochromatin, also had a significant presence in the euchromatin (Figure 2 and 3).

The present results show an uneven chromosome distribution for several motifs within and between the individual chromosomes of *D. melanogaster*. These motifs show a heterogeneous distribution with marked differences in density, both in the euchromatin and heterochromatin. This confirms earlier observations made by *in situ *hybridization showing a high density of SSRs, especially for the mono- and dinucleotide repeats in the euchromatic arm of chromosome X and their absence in chromosome 4, to correlate with the phenomena of gene dosage compensation and recombination [39,40]. The results of the present work suggest that both the absolute and relative frequencies of SSRs recorded to date in the *D. melanogaster *database would be significantly different if the whole-genome sequence were available. The high quantity of GATA repeats in the heterochromatin with respect to the euchromatic regions supports this assumption (Figure 3o-q). Moreover, with the exception of GATA repeats, the remaining SSRs were less abundant in the X than in the Y chromosome. The differences between sexes are notable with respect to some repeats such as AAGAG or AAGAC, which are particularly enriched in the Y chromosome (compare Figure 1b and 1e). Previous molecular characterization of SSRs using total genomic DNA from embryos of *D. melanogaster *indicated that AAGAG and AATAC comprise 5.6%, and 0.52% respectively of the genome [8]. The present ND-FISH results indicate that some SSRs motifs are very abundant in the heterochromatin of *D. melanogaster*, with AAGAG and AATAC the most and least common respectively.

## Conclusions

Our ND-FISH method makes highly efficient the detection of SSRs in *D. melanogaster *chromosomes. The data clearly show the variation in the abundance of different SSR motifs and reveal their non-random distribution within and between chromosomes. As judged by our results, the greater representation of certain SSRs in *D. melanogaster *heterochromatin suggests that its complexity may be greater than previously thought. Thus, the possibility that these sequences may be implicated in some putative heterochromatin role(s) cannot be excluded.

## Authors' contributions

AC performed experiments, design the study and analysed data. AC and NJ wrote the paper. All authors read and approved the final manuscript.
